# Evaluation value of subjective visual quality examination on surgical indications of the early cataracts based on objective scatter index values

**DOI:** 10.3389/fmed.2022.1075693

**Published:** 2022-12-13

**Authors:** Yuzhi Li, Ling Jin, Mingfeng Wu, YuKan Huang

**Affiliations:** Department of Ophthalmology, Tongji Medical College, Union Hospital, Huazhong University of Science and Technology, Wuhan, Hubei, China

**Keywords:** early cataracts, surgical indications, subjective visual function, objective visual function, the objective scatter index

## Abstract

**Aim:**

To evaluate the subjective visual functions of early cataracts patients and assess their surgical indications.

**Methods:**

Eyes were separated into a control group (Group A without cataract) and two early cataracts groups (Group B with 2.0 ≤ OSI < 3.0 and Group C with 3.0 ≤ OSI < 4.0). The objective scatter index (OSI), modulation transfer function cut-off frequency (MTF cut-off), and Strehl ratio (SR) values were applied to measure objective visual functions. The contrast sensitivity (CS) and scores of the questionnaires (QOL and VF-14) characterized subjective visual functions. Above visual functions were compared among three groups. Postoperative visual functions in Group B and C were analyzed to assess the outcome of surgery.

**Results:**

Ninety two subjects (126 eyes) were included in the study. All objective visual function in Group B were significantly better than Group C (all *P* < 0.01), but worse than Group A (all *P* < 0.01). Except for 1.5 c/d CS, subjective visual function in Group A were significantly better than Group B and C (all *P* < 0.05), but there was no significant differences between Group B and C. As for eyes that underwent surgery in Group B and C, all visual functions significantly improved after surgery (*P* < 0.05), except for 1.5 c/d CS in Group C. There were no significant differences among the three groups after surgery.

**Conclusion:**

The subjective visual function can be impaired in early cataracts patients with OSI < 3.0, whose objective visual functions were statistically better than patients with OSI ≥ 3.0. These patients can benefit equally from surgery as patients with OSI ≥ 3.0. Subjective visual functions can be used as surgical indications for these patients.

## Introduction

Cataract is a common eye disease that causes visual function loss due to opacity in the lens. So far, surgery is the only effective way to treat cataracts (1). At present, low visual acuity (VA) is no longer the only indication for cataract surgery. Especially for early cataracts, they still often complain of impaired visual function, even with the good corrected distance visual acuity (CDVA) and only slight lens opacity. In the latest Preferred Practice Pattern (PPP), the visual function is emphasized in the interpretation of cataract surgical indications (2). The analysis of visual function includes two parts, subjectively and objectively. Many studies have found that objective examinations are more reliable and sensitive than subjective examinations (3–5).

Recently, the application of Objective Quality Analysis System II (OQAS II) in guiding surgery for cataract has been widely used (6–9). OQAS II directly collects the retinal images of point light sources through the double-pass system and analyzes their point spread function (PSF) (10). The objective scatter index (OSI) values is calculated by PSF. The OSI values refers to the ratio of the peripheral light intensity to the central peak light intensity of the retinal image, that is, the ratio between the light intensity of the ring area between 12 arc minutes and 20 arc minutes to the light intensity of 1 arc minute (11). The OSI values can be influenced not only by the lens opacities, but also by the tear film instability. And the tear film-related OSI values (TF-OSI) is a quantitative and objective measure of tear-film related vision quality. TF-OSI can excludes the effect of the tear film on the OSI values and it can be calculated by the OSI values and the Mean OSI values. OQAS II provides excellent stability, repeatability, and minimal interference to better assess the actual visual quality of patients (12). A previous study has concluded that OSI ≥ 3.0 may be an objective threshold for preoperative decision-making for cataract surgery (8, 9). According to another research, the OSI equaling to 3.2 was considered as the critical value for surgical treatment (7).

However, there were lots of early cataract patients with CDVA ≤ 0.22 (LogMAR) and OSI < 3.0 that still complained of poor visual quality. This study aimed to explore the visual qualities of such patients and evaluate whether their visual quality could be improved after surgery.

## Materials and methods

### Subjects

**FIGURE 1:**
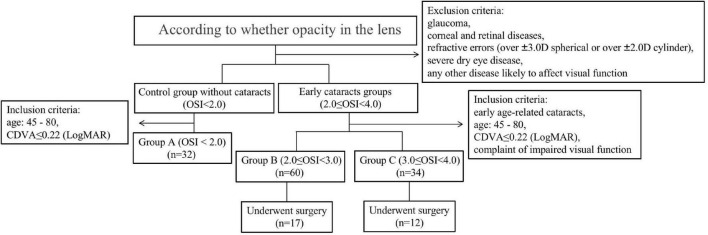
The flow diagram of the overall the study design, including inclusion and exclusion criteria’s as well as participants distribution in each groups.

The study was a prospective, cross-sectional, and self-comparative research. 94 eyes (45 right and 49 left eyes) of 70 patients (24 males and 46 females) diagnosed as early cataracts by the same experienced ophthalmologists from November 2020 to June 2021 at Union Hospital, Tongji Medical College, Huazhong University of Science and Technology were involved. 32 eyes without cataracts (14 right and 18 left eyes) of 22 volunteers (13 males and 9 females) were also enrolled. The main inclusion criteria of early cataract eyes were as follows: early age-related cataracts, age between 45 and 80 years, CDVA of 0.22 (LogMAR) or less, 2.0 ≤ OSI < 4.0, and complaint of impaired visual function. The early cataracts patients with 2.0 ≤ OSI < 3.0 were enrolled in Group B, and the early cataracts patients with 3.0 ≤ OSI < 4.0 were in Group C. The main inclusion criteria of control eyes were as follows: age between 45 and 80 years, CDVA of 0.22 (LogMAR) or less, no lens opacification with OSI < 2.0. The control eyes were in Group A. Patients with glaucoma, corneal, retinal diseases, refractive errors (over ± 3.0 D spherical or over ± 2.0 D cylinder), severe dry eye disease, and any other disease likely to affect visual function were excluded. 29 eyes of 22 patients (17 eyes in Group B and 12 eyes in Group C) underwent cataract surgery (Figure 1). All postoperative evaluations were performed 1 month after surgery until the patients recovered steadily.

This study was approved by the Institutional Review Board of the Ethics Committee of the Union Hospital, Tongji Medical College, Huazhong University of Science and Technology (UHCT20257). All patients have provided written informed consent before participating in this study, and they were examined and treated following the tenets of the Declaration of Helsinki. The clinical trial accession number is NCT04757350.^1^

### Preoperative examinations

Each patient was evaluated by the same ophthalmologist with slit-lamp microscopy to assess the severity of cataracts. Everyone enrolled in this study had refraction, CDVA, intraocular pressure (IOP), and fundus examination. Besides, all people finished the objective and subjective visual function evaluation. Ocular biological parameters and endothelial cell count examination were measured to implant intraocular lens. Each parameter was measured at least three times by the same well-trained doctor.

OQAS II (Visiometrics SL, Spain) test is based on the system setting of the pupil size of 4 mm to ensure consistency. The test was carried out in the darkroom to ensure suitable size of the pupil. And refractive errors are fully corrected during these evaluations: spherical errors are corrected by OQAS II automatically, and cylindrical errors sections are corrected using external lens (12, 13). The double-pass provides three parameters: OSI, Mean OSI, MTF cut-off, and Strehl ratio (SR) (12). Each test was repeated three times for accuracy.

There were two types of CS testing, contrast visual acuity (CVA) and spatial frequency CS. We evaluated the CVA using Binoptometer 4 p (OCULUS, Germany) in a darkroom. By fixing the visual table size (0.4 visual table) and distance (3 m), the operator adjusted the different contrast (80, 40, 25, 20, 15, 10, 7.5, 5%) to measure the CVA of patients, reflecting the ability to distinguish the edges of an object requiring the level of black-and-white contrast. Patients were required to identify the direction of the “E” letter by adjusting the contrast until patients could not recognize the word to achieve the critical value of the CVA. The spatial frequency CS test (SHIQI visual check end) measures the discrimination and physiological function of the human visual system by different spatial frequencies and contrast gratings. This was an important index of ophthalmological disease, which had a weak correlation with visual acuity, and an index of disease progression. It can also help predict visual function (14). Patients were required to identify the direction of the stripe by adjusting the different spatial frequencies and contrast until not being able to recognize the strip. We measured 1.5, 3, 6, 12, and 18 c/d CS. Each measurement was repeated three times per patient.

The Visual Function-14 (VF-14) and Quality of Life (QOL) questionnaires were designed by the American Eye Institute and Aravind Eye Hospital in India. They were often used as an evaluation tool for QOL associated with visual function in cataract patients (15). The VF-14 questionnaire quantifies subjectively the visual function impairments caused by cataracts. This study used the Visual Function Index-14 of Chinese Revision to assess the QOL which is related to subjective visual function of patients (16). QOL questionnaire is also based on daily activities to reflect the QOL affected by the visual function. The patients complete the questionnaire independently under guidance of a same ophthalmologist. Patients first determine whether the daily actions in the questionnaire were limited by the visual function, even with glasses. The degree of difficulty in completing these projects was scored (no difficulty, slightly difficult, very difficult, unable to complete) if the difficulty was caused by decreased visual function. If patients were unable to carry out these activities for other reasons, the item was excluded. Higher scores indicate better visual function (17).

### Surgical technique

All surgeries were done by the same skilled surgeon. Before surgery, the pupil was dilated to 7 mm with 0.5% tropicamide drops (Santen, Osaka, Japan). Phacoemulsification was accomplished under local anesthesia using 0.4% Oxybuprocaine Hydrochloride Eye Drops (Santen, Osaka, Japan). According to preoperative examination results, the 3.0 mm clear corneal incision was located at different positions of corneal limbus in different patients. Continuous circular capsulorhexis was performed, and the hydrodis section and phacoemulsification cataract extraction were performed. Finally, the intraocular lens (ZMB00) was implanted into the capsular bag.

### Postoperative examinations

Postoperative examinations and follow-up visit were routinely performed at 1 day, 1 week, 1 month, 3 month, and 6 month after the surgery. The results of postoperative subjective and objective visual function indexes at 1 month were used for analysis.

### Statistical analysis

SPSS 22.0 and Prism 8 software were used to analyze all data. Continuous variables were expressed as mean ± standard deviation (SD). When parametric analysis was available, ANOVA was performed using Bonferroni *post hoc* analysis to determine significant differences among the three groups. The Kruskal-Wallis tests were used for non-parametric data. Nominal variables were expressed as absolute frequency (*n*) and relative frequency (%). As for sex, eye laterality, and the number of normal CVA eyes, the chi-square test or Fisher test was used to compare the differences among the three groups. Paired *t*-test was performed to compare the continuous variables between preoperative and postoperative parameters in each group if data accords with normality, otherwise Wilcoxon test were used. *P*-values less than 0.05 were considered to be statistically significant.

## Results

### Baseline population data

Total 32 eyes (14 right and 18 left eyes) of 22 volunteers without cataracts (13 males and 9 females) were enrolled in Group A, 60 eyes (28 right and 32 left eyes) of 51 patients (16 males and 35 females) in Group B, and 34 eyes (15 right and 19 left eyes) of 30 patients (11 males and 19 females) in the Group C. The demographics and baseline characteristics of the three groups were similar. No statistically significant differences were found in sex, eye laterality, age, CDVA, and TF-OSI values among the three groups (Table 1, all *P* > 0.05).

**TABLE 1:** Demographics information.

	Group A	Group B	Group C	*P*-value
Age (mean ± SD, years) (range)	66.67 ± 8.90 (51–78)	67.83 ± 7.52 (45–80)	68.03 ± 7.03 (49–80)	>0.05^c^
Laterality (R/L) (relate frequency%)	14/18 (43.8%/56.2%)	28/32 (46.7%/53.3%)	15/19 (44.1%/55.9%)	>0.05^b^
Sex (male/female) (relate frequency%)	13/9 (59.0%/41.0%)	16/35 (31.4%/68.6%)	11/19 (36.7%/63.3)	>0.05^b^
CDVA (LogMAR)	0.07 ± 0.08	0.09 ± 0.08	0.10 ± 0.05	>0.05^a^
TF-OSI (range)	0.38 ± 0.10 (0.20–0.56)	0.38 ± 0.11 (0.10–0.57)	0.38 ± 0.15 (0.11–0.59)	>0.05^a^

CDVA, corrected distance visual acuity; TF-OSI, tear film objective scattering index; LogMAR, logarithm of the minimum angle of resolution.

^a^Kruskal-Wallis test; ^b^Chi-square test; ^c^ANOVN test.

### Objective visual function

The OSI, MTF cut-off, and SR values in the three groups were listed in Table 2. Group B demonstrated lower OSI, higher MTF cut-off and SR values than Group C, meanwhile higher OSI, lower MTF cut-off and SR values than Group A. The differences among the three groups were statistically significant (all *P* < 0.01).

**TABLE 2:** Objective and subjective visual function indexes of the three groups.

	Group A	Group B	Group C	*P*-value
OSI (mean ± SD) (range)	1.0 ± 0.5^+^ (0.2–1.90)	2.3 ± 0.3^+,#^ (2.0–2.9)	3.6 ± 0.3^#^ (3.1–4.0)	<0.01**^a^
MTF cut-off (mean ± SD) (range)	29.14 ± 9.31^+^ (15.63–48.45)	20.55 ± 7.55^+,#^ (4.23–39.29)	13.89 ± 4.93^#^ (8.75–33.48)	<0.01**^a^
SR (mean ± SD) (range)	0.161 ± 0.048^+^ (0.110–0.301)	0.124 ± 0.037^+,#^ (0.050–0.249)	0.098 ± 0.026^#^ (0.071–0.189)	<0.01**^a^
CVA (normal/impaired eyes)(relate frequency%)	21/11^+^ (65.6%/34.4%)	17/43^+^ (28.3%/71.7%)	4/30 (11.8%/88.2%)	<0.01**^b^
1.5 c/d CS (mean ± SD) (range)	51.81 ± 12.61 (22.00–58.00)	45.90 ± 18.27 (9.00–58.00)	47.56 ± 18.50 (9.00–58.00)	>0.05^a^
3 c/d CS (mean ± SD) (range)	72.69 ± 30.72^+^ (7.00–100.00)	43.92 ± 31.98^+^ (5.00–100.00)	43.35 ± 25.65 (5.00–100.00)	<0.01**^a^
6 c/d CS (mean ± SD) (range)	39.72 ± 31.42^+^ (8.00–125.00)	24.68 ± 20.67^+^ (8.00–125.00)	24.35 ± 18.49 (8.00–76.00)	<0.01**^a^
12 c/d CS (mean ± SD) (range)	23.69 ± 14.14^+^ (8.00–62.00)	14.65 ± 8.69^+^ (8.00–58.00)	12.94 ± 7.59 (8.00–40.00)	<0.01**^a^
18 c/d CS (mean ± SD) (range)	15.16 ± 8.43^+^ (1.00–40.00)	10.27 ± 4.85^+^ (8.00–28.00)	8.94 ± 2.42 (8.00–20.00)	<0.01**^a^
Scores of QOL (mean ± SD) (range)	99.05 ± 2.07^+^ (91.67–100.00)	94.53 ± 9.06^+^ (69.44–100.00)	92.50 ± 9.29 (69.44–100.00)	<0.01**^a^
Scores of VF-14 (mean ± SD) (range)	90.76 ± 9.44^+^ (62.50–100.00)	78.56 ± 16.55^+^ (4.17–100.00)	78.98 ± 11.87 (50.00–100.00)	<0.01**^a^

CVA, contrast visual acuity; c/d, cycle per degree; CS, contrast sensitivity; MTF cut-off, modulation transfer function cut-off frequency; OSI, objective scatter index; QOL, Quality of Life; SR, Strehl ratio; VF-14, Visual Function-14.

^a^Kruskal-Wallis test; ^b^Chi-square test; **P < 0.01: compare among the three groups; ^+^*P* < 0.01: compare between Group A and Group B; ^#^*P* < 0.01: compare between Group B and Group C.

### Subjective visual function

There were 21, 17, and 4 eyes with normal CVA (contrast ≤ 25%) in Group A, Group B, and Group C. Accordingly, there are 11, 43, and 30 eyes with impaired CVA (contrast > 25%) in the three groups, respectively (Table 2). The differences among the three groups were statistically significant (*P* < 0.01), but there was no significant difference between Group B and Group C (*P* > 0.05).

**FIGURE 2:**
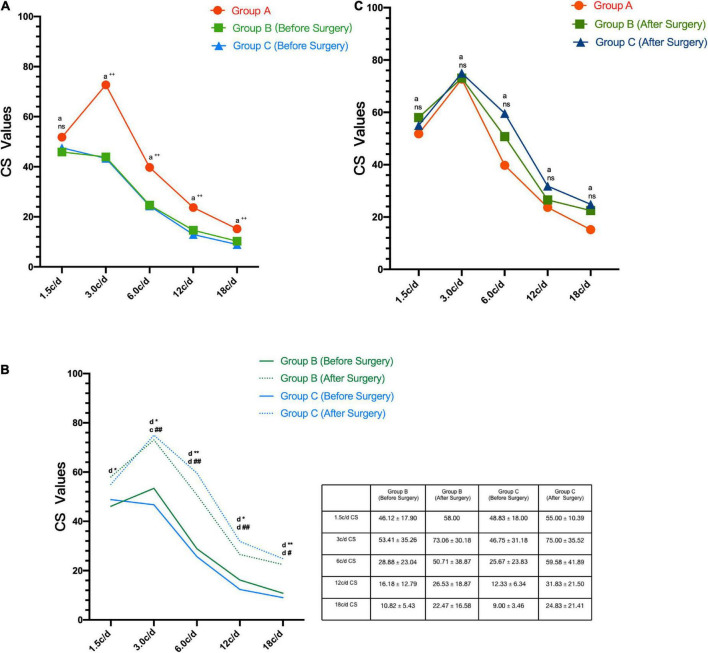
**(A)** CS values at each spatial frequency (1.5, 3.0, 6.0, 12, 18 c/d) in Group A, Group B (Before Surgery), and Group C (Before Surgery) **(B)**. Preoperative and postoperative CS values at each spatial frequency (1.5, 3.0, 6.0, 12, 18 c/d) in Group B and Group C **(C)**. CS values at each spatial frequency (1.5, 3.0, 6.0, 12, 18 c/d) in Group A, Group B (After Surgery), and Group C (After Surgery). (CS, contrast sensitivity; c/d, cycle per degree; ^a^Kruskal-Wallis test; ^c^Paired *t*-test; ^d^Wilcoxon test; ^ns^*P* > 0.05: compare among the three groups; ^++^*P* < 0.01: compare between Group A and Group B; **P < 0.01:compare before and after surgery in the Group B; *P < 0.05:compare before and after surgery in the Group B; ^##^*P* < 0.01:compare before and after surgery in the Group C; ^#^*P* < 0.05: compare before and after surgery in the Group C).

As demonstrated in Table 2, the CS at 1.5 c/d spatial frequencies were 51.81 ± 12.61, 45.90 ± 18.27, and 47.56 ± 18.50 in Group A, Group B, and Group C, respectively. But the three groups did not show significant difference in the CS at 1.5 c/d (*P* > 0.05). As for CS at 3, 6, 12, and 18 c/d spatial frequencies, the control group demonstrated significantly higher CS than the two early cataracts groups (Figure 2A, all *P* < 0.01). But there were no significant differences between the two early cataracts groups in CS at five spatial frequencies (*P* > 0.05).

The scores of the QOL and the VF-14 questionnaires in the three groups were listed in Table 2. There was significant difference in scores of QOL and VF-14 among the three groups (both *P* < 0.01), but not between the two early cataracts groups.

### Comparison of visual quality before and after surgery and postoperative parameters among groups

Seventeen eyes in Group B and 12 eyes in Group C undergone cataract surgery, and all postoperative evaluations were performed 1 month after surgery until the patients recovered steadily. No adverse event occurred.

**FIGURE 3:**
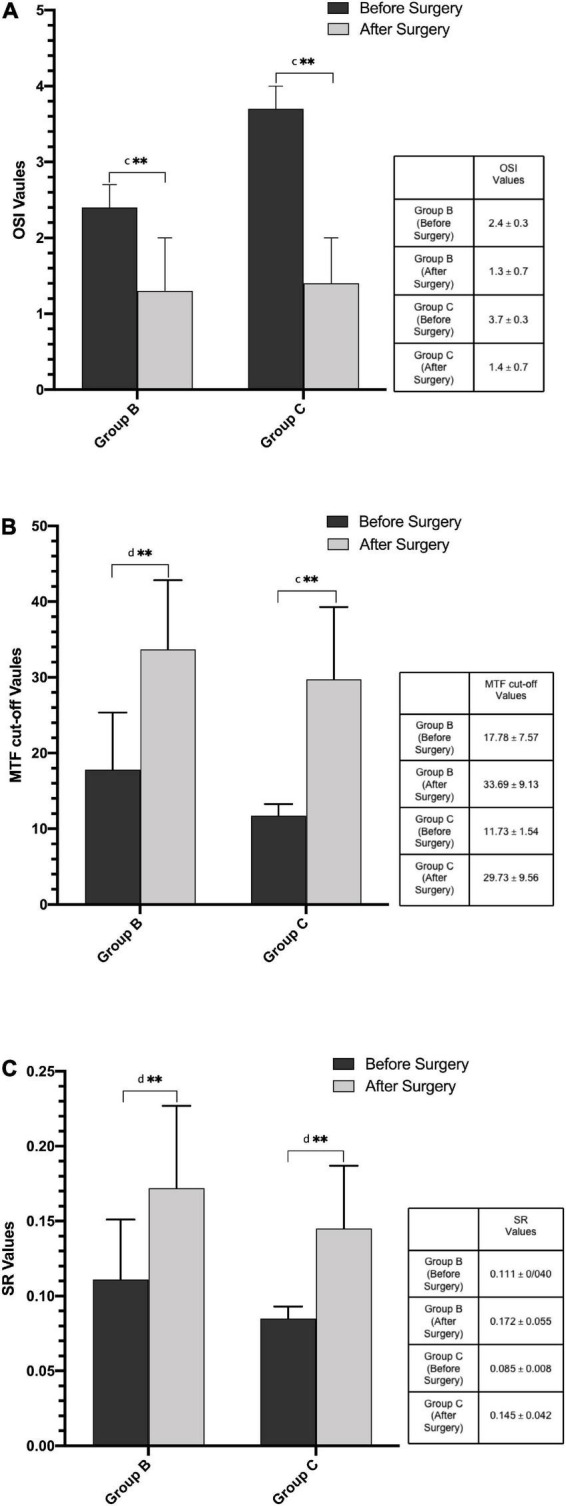
**(A)** Preoperative and postoperative OSI values in Group B and Group C. **(B)** Preoperative and postoperative MTF cut-off values in Group B and Group C. **(C)** Preoperative and postoperative SR values in Group B and Group C. (MTF cut-off, modulation transfer function cut-off frequency; OSI, objective scatter index; SR, Strehl ratio; ^c^Paired *t*-test; ^d^Wilcoxon test; **P < 0.01: compare between the preoperative and postoperative values).

Figure 3 shows the mean preoperative and postoperative OSI, MTF cut-off, and SR values in Group B and C. After the phacoemulsification cataract surgery, objective indexes OSI, MTF cut-off, and SR values improved significantly in the two groups (all *P* < 0.01). There were no statistically significant differences among postoperative parameters in the two early cataracts groups and the control group as for OSI, MTF cut-off, and SR values (Table 3, all *P* > 0.05).

**TABLE 3:** Postoperative objective and subjective visual function indexes of the two groups with early cataracts and these parameters in control group.

	Group A	Group B (after surgery)	Group C (after surgery)	*P*-value
OSI (mean ± SD) (range)	1.0 ± 0.5 (0.2–1.9)	1.3 ± 0.7 (0.3–2.8)	1.4 ± 0.6 (0.4–2.8)	>0.05^c^
MTF cut-off (mean ± SD) (range)	29.630 ± 9.500 (15.630–48.450)	33.690 ± 9.134 (18.620–48.720)	29.730 ± 9.564 (14.890–45.100)	>0.05^c^
SR (mean ± SD) (range)	0.161 ± 0.047 (0.105–0.301)	0.172 ± 0.055 (0.095–0.290)	0.145 ± 0.042 (0.108–0.269)	>0.05^a^
CVA (normal/impaired eyes) (relate frequency%)	21/11 (65.6%/34.4%)	15/2 (88.24%/11.64%)	11/1 (91.67%/8.33%)	>0.05^b^
1.5 c/d CS (mean ± SD) (range)	51.81 ± 12.61 (22.00–58.00)	58.00	55.00 ± 10.39 (22.00–58.00)	>0.05^a^
3 c/d CS (mean ± SD) (range)	72.69 ± 30.72 (7.00–100.00)	73.06 ± 30.18 (14.00–100.00)	75.00 ± 35.52 (7.00–100.00)	>0.05^a^
6 c/d CS (mean ± SD) (range)	39.72 ± 31.42 (8.00–125.00)	50.71 ± 38.87 (10.00–125.00)	59.58 ± 41.89 (8.00–125.00)	>0.05^a^
12 c/d CS (mean ± SD) (range)	23.69 ± 14.14 (8.00–62.00)	26.53 ± 18.87 (8.00–66.00)	31.83 ± 21.50 (8.00–58.00)	>0.05^a^
18 c/d CS (mean ± SD) (range)	15.16 ± 8.43 (1.00–40.00)	22.47 ± 16.58 (8.00–66.00)	24.83 ± 21.41 (8.00–58.00)	>0.05^a^
Scores of QOL (mean ± SD) (range)	99.05 ± 2.07 (91.67–100.00)	99.67 ± 0.92 (97.22–100.00)	99.07 ± 3.21 (88.89–100.00)	>0.05^a^
Scores of VF-14 (mean ± SD) (range)	90.76 ± 9.44 (62.50–100.00)	92.40 ± 7.33 (81.25–100.00)	90.43 ± 9.15 (77.08–100.00)	>0.05^a^

CVA, contrast visual acuity; c/d, cycle per degree; CS, contrast sensitivity; MTF cut-off, modulation transfer function cut-off frequency; OSI, objective scatter index; QOL, Quality of Life; SR, Strehl ratio; VF-14, Visual Function-14.

^a^Kruskal-Wallis test; ^b^Chi-square test; ^c^ANOVA test.

**FIGURE 4:**
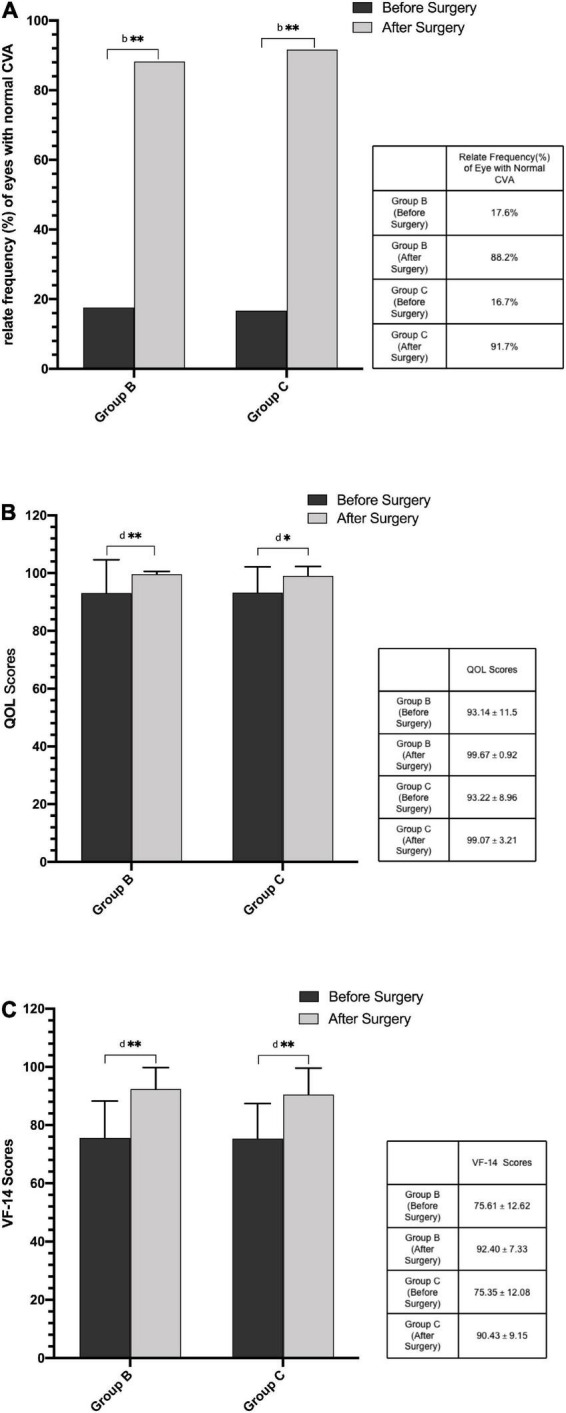
**(A)** Preoperative and postoperative relate frequency (%) of eyes with normal CVA in Group B and Group C. **(B)** Preoperative and postoperative scores of QOL questionnaire in Group B and Group C. **(C)** Preoperative and postoperative scores of VF-14 questionnaire in Group B and Group C. (CVA, contrast visual acuity; QOL, Quality of Life; VF-14, Visual Function-14; ^b^Chi-square test; ^d^Wilcoxon test; **P < 0.01: compare between the preoperative and postoperative values; *P < 0.05: compare between the preoperative and postoperative values).

Figure 4 shows the preoperative and postoperative related frequencies (%) of eyes with normal CVA in Group B and C, and the preoperative and postoperative scores of QOL and VF-14 in Group B and C. The relate frequencies (%) of eyes with normal CVA and the scores of QOL and VF-14 increased significantly after phacoemulsification cataract surgery in the two early cataracts groups (*P* < 0.05). And there were no significant differences between the postoperative CVA and scores of questionnaires in the two groups and these parameters in the control group (Table 3, all *P* > 0.05).

As shown in Figure 2B, the postoperative CS at five spatial frequencies were better than preoperative CS. After the phacoemulsification cataract surgery, the CS at five spatial frequencies improved significantly in the two groups (all *P* < 0.05), except for 1.5 c/d spatial frequency CS in Group C (*P* > 0.05). And there were no significant differences in the postoperative CS at five spatial frequencies between the two groups and the CS in the control group (Table 3 and Figure 2C).

## Discussion

The main discovery of this study is that patients with both impaired objective and subjective visual functions (except for the 1.5 c/d CS), whose OSI was less than 3.0, could benefit from significant visual function improvement after cataract surgeries. Meanwhile, there was no statistically significant differences in the outcomes of surgery between the two groups of early cataract patients with different OSI values. Except for 1.5 c/d CS, subjective visual qualities can be used as a surgical indication for early cataract patients with OSI < 3.0.

The OSI values is currently recognized as a diagnostic parameter capable of discerning surgical cataracts objectively, and as a highly reproducible tool for evaluating optical quality based on the cataract degrades (6, 18–21). Furthermore, more and more researchers proved that the OSI values is the most effective parameter for decision-making in surgery which is approximately 3.0 (9–11). Clinically, many cataract patients with good VA and low OSI values (i.e., less than 3.0), often complained of deterioration of visual quality. As for these patients, the surgical decision-making is more complicated for clinicians.

This research compares the OSI, MTF cut-off, SR, CVA, spatial frequencies CS, QOL and, VF-14 questionnaire together between two groups with early cataracts and the control group without cataracts to evaluate the visual function of early cataracts patients with OSI < 3.0. Moreover, the surgical effects on the two groups with early cataracts were compared to assess surgical indications of early cataracts patients with OSI < 3.0.

We found that MTF cut-off and SR values have significant differences in the three groups, and that there was the highest MTF cut-off and SR values in the control group and the lowest in the group of early cataracts with 3.0 ≤ OSI < 4.0. The MTF cut-off values is the frequency at which the MTF reaches a value of 0.01. SR values is defined as the ratio between the MTF area of the eye to the diffraction-limited MTF area. According to previous studies, the MTF cut-off and SR values decreased significantly with the increase of OSI values (11, 22, 23). This result indicates that the MTF cut-off and SR are equally sensitive based on the OSI grading.

Interestingly, there was a significant difference in CVA among the three groups in the study. However, there was no significant difference between the two early cataracts groups. The result indicated that the early cataract patients with OSI < 3.0 may be affected by the visual disturbance of gray and blurry. In our study, the low spatial frequency CS had no significant difference among the three groups. It is reported that the CS decreased with the increase of scattering for different spatial frequencies (24). This study agrees with the results of the researches, which have concluded that the low spatial frequency CS is of little value in early-stage cataract assessment (25), and that low spatial frequency CS reduced increasingly with late-stage cataract (26). The results of the research showed that the medial and high spatial frequencies CS were better in the control group than the two early cataracts groups, but there were no differences between the two early cataracts groups. This study indicated that the CS at medial and high spatial frequencies had been impaired even though the CDVA and light-scatter were not affected at the earlier cataract stage with OSI < 3.0. Similar to the other studies, the medial and high spatial frequencies CS may be more sensitive than traditional VA tests in quantifying the level of visual damage in early cataract patients (27). Elliott et al. also concluded that CS at high spatial frequency is more sensitive (28). In daily life, light intensity and light contrast are variable. At the same time, we need to identify objects with clear or blurred boundaries. The measurement of central vision underestimates the extent of visual impairment (29). In this study, we measured CS at all spatial frequencies (low, medial, and high) in all subjects with early cataracts and without cataracts. We found that the CS at medial and high spatial frequencies may significantly decrease in early cataracts. The finding suggests that we need to test all spatial frequencies CS, especially the medial and high spatial frequencies CS, to assess the comprehensive visual function of early cataract patients.

The result of our study was that the scores of QOL and VF-14 were the highest in the control group, the lowest in the group of cataracts with 3.0 ≤ OSI < 4.0, which was consistent with the conclusions of previous studies that high OSI levels corresponded with lower VF-14 scores (11, 21, 30). Cataracts in our study were at an early stage with OSI < 3.0, but the scores of VF-14 had decreased. The visual function reflected by QOL and VF-14 questionnaire were critical in understanding and explaining the complaint of patients, and the visual function evaluated by VF-14 had been reported to be a strong indicator of visual quality (31). Although QOL and VF-14 questionnaire is time-consuming and affected by subjective nature, it can also be a decisive test in some uncertain cases, such as early cataracts with good VA and apparent visual disturbances.

In this study, the postoperative objective visual function in the two groups with early cataracts patients were significantly improved. Except for the 1.5 c/d CS in Group C, all postoperative subjective visual function in the two groups with early cataracts were significantly improved. Postoperative parameters of the two groups of cataracts reached a normal level. The results indicated that in the early-stage cataracts patients with good baseline VA, even though the OSI values was less than 3.0, their visual function can be significantly improved through cataract surgeries. Furthermore, the surgical effect on them was the same as cataracts patients with 3.0 ≤ OSI < 4.0.

The OQAS II has recently been used to evaluate the opacity of lens (8, 10, 32). The OSI is an appropriate parameter to objectively distinguish between transparent lens and cataracts, facilitating the decision-making process, particularly in early-stage cataracts (8, 21, 32), with OSI from 3.0 to 7.0 as an indication for surgery (10, 11). In this study, we found that only OSI values cannot explain complaints about the impaired visual quality of early cataract patients or help doctors to decide the timing of surgery. Subjective visual function can verify the symptoms of early cataract patients and guide doctors to decide the timing of surgery.

In this study, our aim was to evaluate the subjective and objective visual functions of early cataracts patients and assess their surgical indications. And we concluded that subjective visual functions can be used as surgical indications for these patients. We have discussed the problems that puzzled many ophthalmologists and patients and reached corresponding conclusions about surgical indications for early cataracts. Therefore this study is of great practical significance. In additions, the samples in the study were examined by slit lamp and divided into early cataracts group and no cataract control group. The devices we used, such as OQAS II, contrast sensitivity, contrast visual acuity tests, as well as VF-14 and QOL questionnaires are common examination in ophthalmology. Therefore, the conclusions of our research can be applied to the ophthalmology departments in other hospitals to a large extent.

There are two limitations to this study. Firstly, large sample size should be provided to obtain more accurate results for reference. Further studies are required to expend the sample size. Secondly, we followed up at 1 week, 1 month, 3 month, and 6 month postoperatively for patients who chose surgical intervention, but only adopted data of 1 month after operation for statistical analysis. In further research, we should conduct statistical analysis of the long-time postoperative data to obtain the change trend of patients’ various visual functions after surgery and judge the long-term effect.

## Conclusion

In conclusion, a combination of current methods, including objective and subjective parameters, should be used for early-stage cataract visual quality evaluating and surgery planning. Subjective visual function indexes can also be used as a meaningful indicator of cataract surgery even the OSI value is less than 3.0.

## Data availability statement

The original contributions presented in this study are included in the article/supplementary material, further inquiries can be directed to the corresponding author/s.

## Ethics statement

The studies involving human participants were reviewed and approved by the Institutional Review Board of the Ethics Committee of the Union Hospital, Tongji Medical College, Huazhong University of Science and Technology (UHCT20257). The patients/participants provided their written informed consent to participate in this study.

## Author contributions

YL, LJ, MW, and YH were responsible for study design. YL, LJ, and MW were involved in date collection and date analysis. YL drafted and wrote the manuscript. YH revised the manuscript. All authors have read and approved the manuscript.

## Footnotes

1
http://www.clinicaltrials.gov
